# Harnessing controlled human infection models to accelerate vaccine development for neglected tropical diseases: Lessons from leishmaniasis

**DOI:** 10.1111/eci.70160

**Published:** 2025-12-17

**Authors:** Vivak Parkash

**Affiliations:** ^1^ Division of Clinical Medicine, School of Medicine and Population Health The University of Sheffield Sheffield UK

**Keywords:** controlled human infection, human challenge studies, *Leishmania*, neglected tropical diseases

## Abstract

**Background:**

Controlled Human Infection Models (CHIMs) offer a powerful approach to expedite vaccine development by enabling early evaluation of vaccine candidate efficacy and immune responses. Their role is increasingly relevant for neglected tropical diseases (NTDs), where traditional trial approaches may be slow, costly, or unfeasible. This review explores the scientific, ethical and translational dimensions of CHIMs, with a focus on the recently developed *Leishmania major* CHIM for cutaneous leishmaniasis (CL).

**Methods:**

A narrative synthesis of peer‐reviewed literature and regulatory guidance documents published over the past two decades was conducted, with additional insights drawn from original fieldwork and the first‐in‐human sand fly‐transmitted CHIM for CL. Considerations included CHIM design principles, immunological outcomes, safety considerations and translational utility, including integration with omics and early phase trials.

**Results:**

CHIMs provide early efficacy data, help identify immune correlates of protection and facilitate the prioritisation of vaccine candidates. The *Leishmania major* CHIM demonstrated safety, high participant acceptability and revealed insights into lesion kinetics and host–parasite interactions. Ethical and regulatory frameworks remain heterogeneous across regions, limiting scalability. Barriers to CHIM deployment in low‐ and middle‐income countries include infrastructure, governance and community engagement challenges.

**Conclusions:**

CHIMs represent a critical translational tool for NTD vaccine research. Lessons from the CL CHIM underscore the potential and challenges of broader implementation. Harmonised regulation, ethical innovation and global collaboration will be essential for future impact.

## Graphical abstract

Controlled Human Infection Models (CHIMs) offer a powerful approach to accelerate vaccine development for neglected tropical diseases (NTDs). This review highlights scientific and translational advances enabled by CHIMs, with a focus on a novel *Leishmania major* model. CHIMs facilitate early efficacy assessment, immune correlates discovery and vaccine prioritisation. Despite ethical and regulatory challenges, particularly in low‐resource settings, CHIMs are gaining recognition by global funders as critical tools to bridge preclinical research and field trials for NTD vaccine pipelines. (Produced using BioRender).

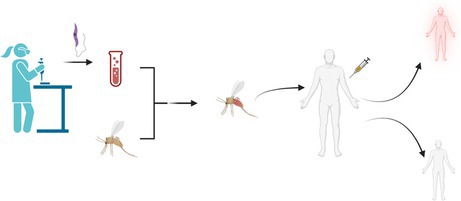

## 1 INTRODUCTION

Neglected tropical diseases (NTDs) affect over 1.6 billion people globally, disproportionately impacting populations living in poverty, conflict and poor sanitation.^1^ Collectively they pose a profound global health challenge. Despite causing significant morbidity and long‐term disability, NTDs such as leishmaniasis, schistosomiasis and Chagas disease remain underfunded in vaccine development pipelines, often falling outside the scope of commercial incentives and conventional research and development (R&D) pipelines. Between 2000 and 2020, only 10% of global infectious disease R&D investments targeted NTDs, with even fewer resources allocated to vaccine development.^2^ This disparity, often in vaccinology, therapeutics and diagnostics, arises from weak commercial incentives, complex transmission ecology and the logistical challenges of conducting efficacy trials in endemic regions.

While effective vaccines exist for diseases like dengue and cholera, most NTDs still lack licensed prophylactic interventions, including leishmaniasis, Chagas disease and schistosomiasis. The resulting reliance on vector control, environmental management and older therapeutics underscores the need for transformative approaches in early phase vaccine evaluation. Controlled human infection models (CHIMs) represent a strategy to address these bottlenecks by generating early efficacy data and mechanistic insights that can strengthen research in diseases traditionally excluded from industrial pipelines.^3^ CHIM studies utilise deliberate infection of fully informed and consented participants to test known and novel therapeutics and vaccines, or to explore immune responses to advance overall understanding of pathophysiology. By deliberately exposing participants to well‐characterised, attenuated, or wild‐type pathogens under tightly controlled conditions, CHIMs enable high resolution investigation of host‐pathogen interactions, early vaccine efficacy signals and immune correlates in a cost and time‐efficient manner. CHIMs have already revolutionised vaccine development for malaria, typhoid and cholera, and their strategic extension into NTDs represents a major opportunity for accelerating global health innovation. Another major bottleneck in NTD research is the absence of reliable correlates of protection, coupled with the inherent difficulty of conducting large‐scale efficacy trials particularly in resource‐limited, endemic settings. It should be noted that these issues can also occur in well‐funded research settings, reflecting the overall lack of activity in the field of NTDs.^4^ CHIM studies have been shown to impact these areas, despite being outside of the original scope of many CHIM studies.^5^, ^6^, ^7^ Although there is an awareness of this need to develop novel vaccines, only 1 NTD (*Leishmania*), has been named by WHO as an endemic pathogen for which new vaccines are urgently needed.^8^ *Leishmania* however has at least been recognised as the highest priority parasite target for novel vaccines after *Plasmodium falciparum* malaria by WHO.^9^

This narrative review draws on lessons from the development of the world's first CHIM for *Leishmania major*,^9^ exploring its design, implementation and implications for broader translational science. This article examines how CHIMs can be ethically and scientifically applied to other NTDs, assesses their role in modern vaccine development frameworks and outlines a roadmap for responsibly expanding their use in both high‐ and low‐resource settings. Through the lens of leishmaniasis, the potential for CHIMs to close long‐standing gaps in vaccine development for many NTDs with similar profiles is considered.

## 2 METHODS

This is a narrative review using PubMed with the inclusion of relevant research articles using the following terms: ‘Controlled human infection’, ‘Human challenge’, ‘neglected tropical diseases’, ‘experimental infection’, ‘volunteer infection study’, ‘challenge agent’. The search results were then filtered based on their titles. Further filtering subsequently took place by review and evaluation of the abstract. Results published since 2000 were prioritised; although additional articles linked to these publications (such as through citation) were further identified. In all cases the full text of the article was reviewed.

## 3 CONTROLLED HUMAN INFECTION MODELS: PRINCIPLES AND APPLICATIONS

While ethically complex, CHIMs have a long‐standing history in biomedical research, stretching back to the 18th and 19th centuries when early inoculation studies were performed for diseases like smallpox and cholera. In the 20th century, these models played a pivotal role in the development of vaccines against typhoid, influenza and malaria. However, their use became more limited in the latter half of the century due to significant ethical concerns and a history of harmful practice, as well as the rise of animal models. In recent decades, however, there has been a notable resurgence in the interest and application of CHIMs, driven by growing recognition of their unique value in accelerating vaccine development and improving understanding of host‐pathogen interactions but also increased efforts to ensure ethical practice with fully informed consent and public engagement. CHIMs now exist for many infectious diseases, described in detail elsewhere.^7^, ^10^, ^11^, ^12^, ^13^, ^14^, ^15^, ^16^, ^17^, ^18^, ^19^, ^20^, ^21^

The renewed momentum for CHIMs coincides with a shift in vaccine development, particularly in the wake of the COVID‐19 pandemic.^11^ As expectations for a rapid response to emerging pathogens grow, CHIMs have emerged as an important translational tool. When conducted under rigorous ethical and regulatory oversight, CHIMs can complement traditional preclinical and later phase studies, providing a more patient‐focused perspective that is often lacking in animal models. They allow researchers to examine disease progression, immune dynamics and treatment efficacy in a reproducible, and often mechanistically informative environment.

One of the primary scientific advantages of CHIMs lies in their capacity to elucidate immunological correlates of protection. These are the specific immune responses that predict whether an individual is protected from infection or disease. Identifying these correlates remains a major barrier in the development of many vaccines, particularly for neglected tropical diseases (NTDs), where naturally acquired immunity may be partial, and effective animal models are limited or absent.^7^ CHIMs also support the early stage evaluation of vaccine candidates, enabling up‐selection of promising candidates and early down‐selection of ineffective or unsafe ones before committing to costly and logistically challenging field trials. This not only reduces the risk of failure in late phase trials but also ensures that resources can be redirected more efficiently across a broader pipeline, increasing the overall likelihood of successful vaccine development.

However, the predictive validity of CHIMs must be interpreted carefully. In some cases, the artificial nature of infection (e.g. pathogen strain, route or dose) may differ significantly from real‐world exposure in endemic settings. Additionally, participants are often healthy, immunocompetent adults in high‐income countries, which may limit generalisability to vulnerable populations, such as children or individuals with comorbidities. Despite these limitations, CHIMs offer a powerful tool for reducing uncertainty and gaining mechanistic insights early in the translational pathway.^7^ A growing number of CHIMs have already made substantial contributions across a range of pathogens. For example, a CHIM for *Streptococcus pneumoniae* demonstrated that nasal colonisation with pneumococcus induces protective immunity against subsequent carriage, a finding that could not be reproduced in animal models. Moreover, this study quantified the dose‐dependent nature of antibody responses, providing critical data for vaccine development.^12^

In norovirus CHIMs, researchers found that immunocompetent adults do not develop detectable viraemia, challenging prior assumptions about systemic dissemination. Archived samples from this model have since been used in newer studies to improve diagnostic assays and inform vaccine development strategies.^13^ Similarly, CHIMs for schistosomiasis have helped validate early diagnostic tests, even though the short‐term infection protocol did not fully replicate the effects of chronic or repeated exposure typical of endemic regions.^14^ In the field of dengue virus research, CHIM studies have yielded insights into pathogenesis and immune memory that could not be captured in studies on non‐human primates, which fail to exhibit clinical disease despite viral replication.^7^ These models have provided valuable longitudinal samples for biomarker discovery and the identification of protective responses. Similarly, a malaria CHIM revealed that robust T‐cell responses, though detectable, did not correlate with reduced parasite growth rates, reinforcing the importance of humoral immunity and guiding focus towards antibody thresholds in candidate evaluation.^15^

Perhaps most notably, CHIMs are now being considered for diseases thought to be previously unsuitable owing to presumed ethical, technical and microbiological barriers. This includes experimental models using Bacillus Calmette–Guérin (BCG) as a proxy for *Mycobacterium tuberculosis*,^16^ and the development of a SARS‐CoV‐2 CHIM,^10^ which has attracted global attention for its potential to streamline efficacy testing during pandemics. These emerging applications highlight how improved containment strategies, refined ethical frameworks and community engagement are expanding the feasible boundaries of controlled human infection research. The discussion has now turned to diseases with higher patient consequence including plague^17^ and Nipah virus.^18^

CHIMs represent a re‐emerging yet contemporary method of investigating infectious diseases. By enabling controlled studies of infection, immunity and intervention, they offer strong translational value particularly in diseases where traditional models are lacking. Their strategic use is becoming an important consideration in modern vaccine development, especially for NTDs and associated diseases, where market‐driven innovation is limited, and public health urgency is high.

## 4 CUTANEOUS LEISHMANIASIS: A CASE STUDY FOR CHIM

Cutaneous leishmaniasis (CL), a disfiguring skin disease caused by *Leishmania* parasites, represents a significant public health challenge in endemic areas. However vaccine development has lagged behind due to a limited understanding of protective immunity and a lack of validated endpoints.^1^, ^2^ The resulting scarring can be disfiguring and can result in a significant psychosocial impact.^19^, ^20^, ^21^ The development of a CHIM for *Leishmania major*, the principal cause of zoonotic CL in ‘Old World’ regions (predominantly Africa and Asia), addresses this gap by enabling direct study of host–pathogen interactions and the potential for early‐stage vaccine testing. CL is endemic in around 90 countries reporting to the WHO and 200,000 new cases were reported in 2020, which is thought to be a fraction of the total burden.^3^ CL can present as an inflammatory lesion, usually at the bite site but the range of disease is dependent on the species involved. *Leishmania major*, commonly self‐resolves over a period of months and has recognised topical treatments. This usually results in solitary lesions.

Despite extensive preclinical research over several decades, progress in developing a vaccine for leishmaniasis remains limited.^12^, ^13^ Numerous candidates have been evaluated in animal models, yet few have progressed to clinical trials.^8^ This stagnation in the vaccine pipeline is attributed to multiple factors, including the complexity of host–parasite interactions and gaps in understanding human immune responses.^12^ Recent studies have begun to quantify both the global demand and the affordability of a successful leishmaniasis vaccine, underscoring the urgency of accelerating development.^10^, ^11^ To overcome existing barriers, there is a clear need for novel approaches that facilitate the identification and validation of vaccine candidates, and enhance understanding of both natural and vaccine‐induced immunity in humans.

CL exhibits substantial variability in clinical presentation and immune response, with natural immunity typically acquired following infection and healing. Observational and animal model data suggest that recovery from CL confers robust long‐term protection, particularly against homologous reinfection. This characteristic, combined with the self‐limiting nature of many *L. major* infections, makes CL a suitable candidate for human challenge. Given that arguably the most important factor in the paradigm for a suitable CHIM development includes the knowledge of a treatable strain with relevance to human disease,^3^ *L. major* is a strong candidate. Given also the genetic homogeneity between species and the knowledge of heterologous protection against species causing visceral disease, this increases the use case for *L. major* within CHIMs and subsequent relevance to vaccine testing.^22^, ^23^ Furthermore, vaccine efforts have long been hampered by the absence of clear immune correlates of protection. CHIMs provide the opportunity to derive mechanistic insights into protective immunity, enabling immunological endpoints to be defined and used in vaccine down‐selection. In developing a CHIM for CL, a number of enabling studies were conducted: a public involvement exercise to understand the need and public perception of a potential CL CHIM, development of a novel parasite bank, a study using uninfected sand flies to determine the protocol and the most favourable vector. The final phase was the culmination of this work resulting in the *Leishmania* human challenge (CHIM) study. Throughout this body of work, focus groups were conducted to check acceptability.

Given the novel and ethically complex nature of deliberately infecting healthy individuals with a pathogen that can lead to scarring, the project began with a comprehensive review of public and stakeholder opinions. This included a public involvement group with lay members of the public, patient representatives and individuals who had undergone CHIM studies for other diseases.^24^ Participants were invited to discuss their perspectives on deliberate infection, scarring, compensation, consent and risk. These discussions suggested conditional acceptability: participants were supportive provided that risks were well communicated, treatments were readily available, and cosmetic outcomes were monitored and supported. Feedback from these engagements directly informed the development of the study's participant information materials, consent forms, risk mitigation strategies and overall design. The most important outcome from this activity was the evaluation of the use of excision biopsy to terminate infection. This method was universally favoured by public involvement participants due to both the altruistic aspect of donating tissue that could lead to further scientific discovery and novel insights, but also the psychological aspect of removing parasitic material.

The first clinical study, FLYBITE, focused on assessing the feasibility and safety of sand fly exposure in humans using uninfected *Phlebotomus papatasi* and *P. duboscqi*, two natural vectors o*f Leishmania major*.^25^ Conducted at a UK clinical research facility (University of York), healthy adult participants were exposed to five female sand flies within a custom made, watch‐like, feeding chamber secured to the forearm. Outcomes included successful blood feeding rate, participant tolerance, adverse events and acceptability. There was no significant difference between the bite rate of the 2 vectors, *P. papatasi* and *P. duboscqi* on participants exposed to 5 sand flies (3.3 ± 0.81 vs. 3.00 ± 1.27 bites per participant; *p* = 0.56). The study demonstrated that controlled exposure to sand fly bites was feasible and safe in a clinical environment. Most participants experienced only mild, transient local skin reactions and no serious adverse events occurred. Participant feedback via focus group indicated high levels of comfort, trust in the team and willingness to participate in future infected studies. These findings provided a crucial proof‐of‐concept that sand fly transmission could be managed ethically and safely in a controlled research setting in a non‐endemic country.

A critical factor in selecting an infectious agent for use in CHIMs is full traceability and safety of the challenge agent. Ensuring the provenance of the challenge agent mitigates the risk of inadvertent transmission of blood‐borne infections, including viruses, bacteria, parasites and prions, and guards against contamination from in vitro exposures to potentially hazardous agents, such as bovine derived materials associated with transmissible spongiform encephalopathies (TSEs). While numerous parasite banks offer *L. major* isolates none currently meet all the stringent requirements under good manufacturing practice (GMP) conditions, particularly if used in the future as an investigational medical product (IMP). In addition, concerns remain regarding the poorly understood leishmaniaviruses and similar viruses which may exert species‐specific or even strain‐specific effects.^26^, ^27^ There is emerging evidence that some of these viruses may be acquired through arthropod vectors, raising concerns about their possible classification as arboviruses.^28^, ^29^ Consequently, the use of parasites from non‐human or poorly characterised sources presents scientific and ethical uncertainties. Moreover, the potential for genetic drift, intra‐strain variability or hybridisation is particularly concerning when working with isolates derived from non‐clinical samples.^30^, ^31^ Therefore, for CHIM use, it is essential that the challenge strain be well characterised, genetically stable and isolated directly from a confirmed clinical case. In response to this need, our team led the development of a novel GMP‐compatible *L. major* parasite bank at the University of York.^32^

Parasites were freshly isolated from consenting patients with confirmed cutaneous leishmaniasis (CL) at the Department of Medicine, Chaim Sheba Medical Center, Israel. One isolate, designated L. major_MRC‐02, was obtained under aseptic conditions from a patient who presented with two ulcerated papules on the leg and a nodule on the neck approximately 5 months following exposure to sand flies in the Negev region. The patient elected not to receive treatment, and the lesions healed spontaneously within 4 months. Eighteen months later, follow‐up confirmed sustained healing with minimal residual scarring. The patient tested negative for HIV, HBV, HCV and HTLV‐1. Primary parasite culture was established at passage 1 using GMP‐grade media at the Hebrew University. Foetal calf serum was sourced from a TSE‐free certified supplier (ThermoFisher). Cryopreserved master stocks were maintained in liquid nitrogen and distributed to York and Prague for characterisation, and to Vibalogics (Cuxhaven, Germany), a Contract Development and Manufacturing Organisation (CDMO), for GMP banking. Comprehensive genomic characterisation was performed through next‐generation sequencing (NGS) at Genome Québec, using DNA extracted at the University of York. Additional DNA was sequenced following passage in BALB/c mice. Phylogenetic and comparative analyses demonstrated: (i) the isolate's identity as *L. major*, consistent with a likely Israeli origin; (ii) several single nucleotide polymorphisms (SNPs) relative to the Friedlin reference strain; and (iii) genetic stability after a single murine passage, with no significant sequence or copy number variation.

The final GMP‐compliant parasite bank is of sufficient scale to support CHIM studies in over 1200 individuals, including those involving sand fly transmission. While the number of challenge experiments that can be performed using this isolate is inherently limited, the production process prioritised minimising in vitro expansion to preserve genetic and phenotypic integrity. The resulting GMP‐grade L. major_MRC‐02 strain is therefore also suitable for use in needle‐based challenge models, in compliance with investigational medicinal product (IMP) regulations.

The choice of the *Leishmania major* parasite and the *Phlebotomus duboscqi* sand fly vector was guided by biological and clinical considerations following confirmation of the above initial studies. *L. major* is dermotropic and typically causes self‐limiting, localised and non‐visceralising disease in immunocompetent individuals, making it a suitable candidate for human challenge. *P. duboscqi* is a proven vector, maintainable in insectaries and capable of consistent infection with laboratory strains. Together, this combination provides a relevant and biologically faithful model of natural transmission, which had never previously been achieved in a human challenge setting.

**FIGURE 1:**
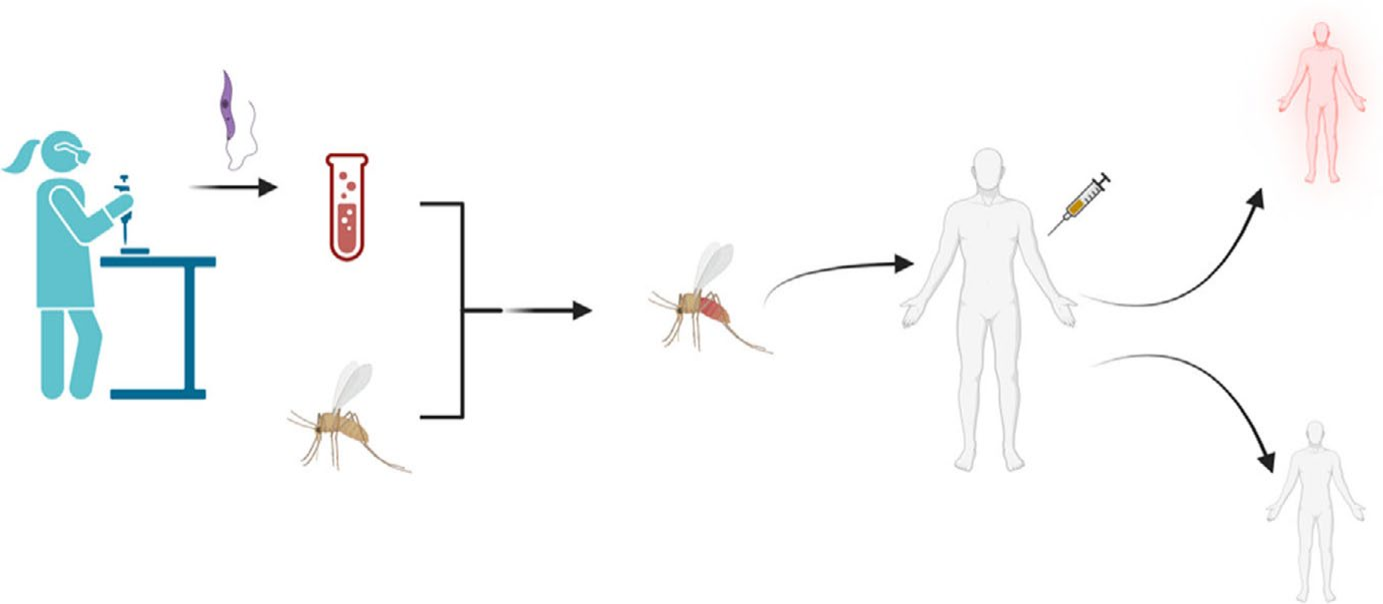
(Created in BioRender). Overview of *Leishmania* controlled human infection. A clinical isolate from a named patient is obtained and analysed. *Leishmania* promastigotes are then used to infect blood on which sand flies are fed. Infected sand flies are then exposed to consented participants, who have already received the candidate vaccine. After reviewing the number of participants who develop the disease, vaccine efficacy can then be determined.

Following the success of FLYBITE, the next phase involved infecting *P. duboscqi* with *L. major* promastigotes (from our parasite bank) and using these to challenge participants. The infection protocol involved feeding sand flies on blood meals infected with *Leishmania*. Infection status and metacyclic load were confirmed through dissection and quantitative PCR of a proportion of the infected colony. A stringent clinical protocol was developed, including exclusion of participants with immune compromise, prior *Leishmania* exposure, and dermatological risk factors. 14 participants were exposed to 5 infected sand flies, again via a feeding chamber. Participants were monitored for 4 hours post‐exposure in the clinical facility, followed by weekly outpatient visits for 12 weeks and monthly visits thereafter for up to 1 year. Lesion development was observed in the majority of participants (over 85%), with onset occurring between 10 and 42 days post‐challenge. Lesions were typically erythematous papules, with a proportion progressing to shallow ulceration. Diagnosis was confirmed via skin biopsy, microscopy, PCR and culture. Histological examination revealed classic features of CL, including granulomatous infiltrates, amastigote‐containing macrophages and epidermal hyperplasia. Lesions were monitored for size, ulceration, pain and cosmetic impact. Treatment was primarily by use of excision biopsy. 3 participants had recurrence of disease, which was successfully treated with cryotherapy. Recurrence was consented for as a possible outcome of the process in the initial consent process. Although scarring was seen to varying degrees with all participants and was consented for, it was noted that scarring was higher with recurrence with commensurate participant acceptability. All participants responded to therapy and completed the study with no serious adverse outcomes.^9^ Figure 1 demonstrates how this model can be used to determine candidate vaccine efficacy.

Initial analysis has revealed an early expansion of activated CD4+ T cells producing IFN‐γ and TNF‐α in response to soluble *Leishmania* antigen stimulation. These cytokines, which are central to macrophage activation and parasite killing, increased significantly by 2 weeks post‐exposure in most participants. Additionally, polyfunctional T cell populations, co‐expressing IFN‐γ, IL‐2 and TNF‐α, were identified, correlating with lesion size and resolution. Transcriptional profiling of lesions at different stages demonstrated a shift from pro‐inflammatory responses towards regulatory and wound‐healing signatures over time. These findings support the hypothesis that successful resolution of CL requires both parasite control and immunomodulation to limit tissue damage. Many of these insights have been learnt through the use of novel techniques such as spatial transcriptomics.

The translational implications of this CHIM are substantial. The model provides a novel platform for both up‐ and down‐selection of vaccine candidates prior to field deployment. This is particularly valuable in leishmaniasis, where field trials are expensive, prolonged and require large cohorts. Using the CHIM, vaccines can now be evaluated for efficacy under conditions that mimic natural transmission, including vector delivery, skin inoculation and host‐pathogen‐vector interactions. The model allows direct comparison between immune responses induced by vaccination and those generated during natural infection, facilitating the identification of correlates of protection. It also provides an opportunity to evaluate immune memory, cross‐species protection and boosting effects in a controlled setting.

Beyond vaccines, the CHIM offers a testing platform for novel therapeutics and topical treatments. It can be used to evaluate the efficacy, safety and pharmacodynamics of skin‐directed therapies, including immunomodulators and topical agents. In addition, digital imaging and emerging wearable technologies can be trialled in parallel to refine clinical endpoints and monitoring tools. Understanding early immune responses to *Leishmania* infection is key to identifying the determinants of protection versus pathology. In future studies, it could be adapted to explore co‐infections, host genetic variability, or age‐related differences in immune response. Combined with systems immunology approaches, the CHIM has the potential to uncover new mechanisms of pathology and resolution.

## 5 TRANSLATIONAL IMPACT OF CHIMs ON VACCINE DEVELOPMENT

CHIMs offer a potentially transformative platform for vaccine development, particularly for neglected tropical diseases (NTDs) where traditional pipelines are inconsistent. The ability to assess candidate vaccine efficacy, immune responses and pathogen‐host dynamics in a controlled setting underpins their utility. CHIMs provide a unique opportunity to detect early signals of vaccine efficacy, enabling researchers to up‐select promising candidates and down‐select those lacking protective efficacy before expensive field trials. This is particularly relevant for NTDs, where trial infrastructure may be limited, and endpoint variability complicates interpretation. This translational pipeline helps prevent poorly performing candidates from progressing to large‐scale trials, conserving both financial and logistical resources.

Modern CHIMs generate rich longitudinal datasets, integrating immunomonitoring tools such as flow cytometry, transcriptomics, proteomics and spatial tissue profiling. This integration facilitates high‐resolution mapping of immune correlates of protection, critical for NTDs like leishmaniasis and schistosomiasis, where such correlates remain undefined. In the *Leishmania* CHIM, lesion biopsies and serial blood samples revealed type I interferon signatures and recruitment of CD8+ T cells that did not necessarily correlate with faster lesion healing, highlighting the complexity of protective immunity. These data inform not only vaccine design but also assay development for downstream trials.

CHIMs act as a bridge between preclinical work and field trials, addressing critical uncertainties that frequently derail NTD vaccine development. By defining correlates of protection and establishing proof‐of‐concept efficacy, CHIMs reduce the need for large‐scale, high‐risk phase III trials for all promising candidates. Compared to field trials, CHIMs are faster and more cost effective, often involving fewer than 50 participants and producing results within months. They also allow for simultaneous testing of multiple vaccine candidates or regimens. A CHIM for schistosomiasis, for instance, enabled early‐stage validation of diagnostic tools and screening of vaccine‐induced immune responses. This would have otherwise required extensive field infrastructure and years of follow‐up. For diseases like Chagas and dengue, where field trial costs are challenging and incidence rates fluctuate, CHIMs may offer a pragmatic alternative to gain early insights. This accelerated time to vaccine deployment, the opportunity to de‐risk development, and provide mechanistic insights that are unattainable in animal models or natural history studies can be attractive to funders. Their strategic application in NTD vaccine development holds promise for breaking the cycle of limited funding oppurtunities and delivering timely interventions to underserved populations.

## 6 ETHICAL, REGULATORY AND GLOBAL EQUITY CONSIDERATIONS

The development of CHIMs for neglected tropical diseases (NTDs), exemplified by the *Leishmania major* CHIM, raises complex ethical, regulatory and global equity considerations that must be addressed. As CHIMs transition from proof‐of‐concept studies in high‐income countries to broader application in endemic settings, rigorous governance, community engagement and collaboration become paramount.

The *Leishmania* CHIM was designed in tandem with a robust ethical framework to ensure acceptability, safety and transparency. Ethical approval was granted by the UK Health Research Authority as well as the institution at which the study took place. The study was registered in international clinical trial databases. Participants were provided with detailed, informed consent procedures, dermatological and psychological assessment and long‐term follow‐up to monitor clinical and psychosocial outcomes. Compensation was carefully benchmarked to reflect time commitment and inconvenience. Crucially, participants were involved throughout the process, with feedback mechanisms integrated into study procedures.

While the *Leishmania* CHIM was conducted in a high‐resource setting, a core objective is eventual adaptation for use in endemic low‐ and middle‐income countries (LMICs). Such expansion poses ethical and logistical challenges, particularly when populations may have distinct vulnerabilities ranging from limited healthcare access and pre‐existing conditions to socioeconomic disadvantage. Differences in immune status, skin microbiota and prior sand fly exposure may also alter disease trajectories or immune responses, impacting generalisability and risk profiles. A phased, partnership‐based approach will be critical. By collaborating with endemic‐region institutions and investing in joint capacity building, CHIM infrastructure can be responsibly adapted for local contexts. Ethical strategies including fair compensation, community consultation and participant protections must be revisited to reflect local standards, norms and risks. This approach helps guard against ethical dumping, where high‐risk research is relocated to jurisdictions with weaker regulatory safeguards.^33^ Public involvement will therefore be crucial to ensure an ethical and collaborative process.

The regulatory landscape governing CHIMs varies substantially across countries and is closely tied to the nature of the study and setting in which it is conducted. In the United Kingdom, studies involving an Investigational Medicinal Product (IMP), such as a novel vaccine, fall under the jurisdiction of the Medicines and Healthcare products Regulatory Agency (MHRA).^34^ However, the infectious challenge agents used in CHIM studies such as pathogens administered to elicit controlled infection are not currently classified as IMPs. Consequently, CHIM protocols that do not involve a licensed or investigational drug product, including those involving *Leishmania major* alone, do not require MHRA approval for the challenge component per se. If, however, a CHIM study incorporates an IMP, such as a vaccine candidate, then the MHRA assumes responsibility not only for the IMP but also for assessing the risk–benefit profile of the proposed challenge model. In such cases, the challenge agent is reviewed in the context of the broader investigational framework. Regardless of MHRA classification, it is standard practice in the UK to ensure that CHIM studies conform to Good Clinical Practice (GCP) guidelines, including participant safety, data integrity and quality assurance. Internationally, regulatory requirements differ markedly. In the United States, for instance, challenge agents may be classified and regulated as biologics or investigational products, necessitating formal regulatory authorisation and manufacturing under Good Manufacturing Practice (GMP) conditions.^18^, ^35^ In contrast, some low‐ and middle‐income countries (LMICs) lack clear regulatory frameworks or specific guidelines for the conduct of CHIMs. In such settings, investigators are encouraged to engage proactively with national regulatory authorities overseeing IMPs or clinical trials to seek guidance and ensure adequate oversight.

CHIMs have the potential to facilitate and support regulatory approval and licensure. Although their main use is to demonstrate the potential for novel antigens and vaccine candidates, they can also rarely be used directly to inform licensure. Following a vaccine‐CHIM for *Vibrio cholerae*, the US Food and Drug Administration (FDA) licensed the novel Vaxchora vaccine for use in travellers to areas where the risk of cholera was significant.^36^ Following a vaccine‐CHIM for typhoid, results demonstrated efficacy and paired with immunogenicity data, this resulted in WHO prequalification for use in endemic regions.^37^

To address these disparities, the World Health Organisation (WHO) issued a position paper in 2016 on the conduct and regulation of CHIM studies.^38^ The document outlines key considerations, including the provenance of challenge agents, scientific justification for controlled human infection, risk minimisation strategies, infrastructure requirements and ethical oversight mechanisms. The WHO's guidance serves as a valuable template for countries without established CHIM‐specific legislation and promotes consistency across global jurisdictions. Despite these efforts, regulatory and clinical endpoint harmonisation remains an unmet need. Differences in how challenge agents are classified, what constitutes appropriate risk mitigation, and the extent of ethical review can significantly influence the feasibility of multicentre CHIMs or their translation into endemic settings. For example, the establishment of a CHIM for schistosomiasis in Uganda highlighted critical regulatory and governance challenges, including gaps in national capacity to assess novel trial designs, biosafety standards and participant protections.^39^, ^40^

Public and community engagement are essential to the ethical and long‐term sustainability of CHIMs. In the *Leishmania* CHIM, proactive public involvement shaped both study design and communication strategies. Participants were consulted about lesion site preferences, compensation and post‐trial follow‐up, enhancing acceptability and transparency. Building similar engagement models into CHIM deployment in endemic regions will require culturally tailored approaches, early involvement of local stakeholders and partnerships with patient‐public representatives. Effective engagement fosters trust, dispels misinformation and enhances research literacy. It also facilitates ethical oversight and shared accountability by embedding public values into trial governance. For NTDs, which often carry stigma and affect marginalised communities, inclusive engagement is particularly critical. Given the skin manifestations caused by CL, there have been several parallels made with other skin NTDs such as Buruli Ulcer. The development of a CHIM for Buruli ulcer was influenced by the CL stakeholder engagement process. It was assumed that this CHIM would not be acceptable in an Australian setting, however public and community engagement suggested the acceptability and potential utility of such a CHIM.^41^ This reinforces the need to share lessons learnt in CHIM studies, which organisations such as HIC‐Vac are championing.^42^

CHIMs for vector‐borne diseases like leishmaniasis demand specialised entomological and laboratory capabilities. Maintaining sand fly colonies, validating parasite loads and ensuring transmission fidelity require expertise and containment infrastructure not yet widely available in endemic areas. However, these barriers are not insurmountable. By investing in training, laboratory upgrades and knowledge exchange, CHIM capacity can be expanded to LMIC institutions with existing NTD research platforms. This aligns with broader goals of decolonising global health research and fostering equitable Global North–South collaborations.

Whilst CHIMs can aid in the development of vaccine candidates, they have several limitations. CHIMs typically involve a small cohort of volunteers, which restricts their capacity to identify rare adverse events or generate precise estimates of vaccine efficacy. This limitation is especially pertinent for neglected tropical diseases, where pharmacovigilance systems in low‐ and middle‐income countries may be limited or underdeveloped. Population selection in general remains challenging. Given that measured vaccine efficacy can differ between populations, there are renewed efforts to conduct CHIMs in endemic settings, although this remains limited.^18^, ^43^ CHIMs are largely conducted in healthy volunteers, and avoid paediatric, immunocompromised and pregnant populations led both by ethical and regulatory concerns. These populations are sometimes disproportionately affected by some NTDs, and so exclusion may result in fundamentally different immunological outcomes. In the case of leishmaniasis, where malnutrition, chronic illness and cumulative exposure are common in endemic regions, the biological relevance of findings derived from healthy volunteers is inherently limited and raises the question of translation to real‐world populations. CHIMs also employ a well‐characterised challenge agent, which has often undergone significant testing, selection and sometimes passage prior to using the strain. This can maximise reproducibility and safety, but may diverge from the response to natural exposure, where for example multiple strains of a pathogen can be inoculated at exposure. For vector‐borne diseases, it is therefore paramount to consider the impact of the vector in transmission models.

The *Leishmania* CHIM demonstrates that ethically sound, participant‐centred and scientifically rigorous CHIMs can be developed even for disfiguring, vector‐borne diseases. These lessons must now inform efforts to scale CHIMs for other NTDs and deploy them more equitably. The importance of capacity building in endemic settings, and embedding public involvement to safeguard participant welfare, will ensure that CHIMs contribute not only to vaccine development but also to trust in global health research.

## 7 BROADER POTENTIAL IN OTHER NTDs

While cutaneous leishmaniasis represents a compelling example, CHIMs could have broader applications across other high burden NTDs if technical, ethical and operational challenges can be overcome. CHIMs for schistosomiasis have already progressed, using low‐dose cercarial exposure in healthy participants to assess immune responses and test diagnostics. Dengue virus CHIMs, despite the risk of antibody‐dependent enhancement, have yielded important immunological data and are informing vaccine development in endemic and non‐endemic regions. Hookworm CHIMs have facilitated early vaccine trials focusing on adult‐stage challenge following larval exposure. For Chagas disease, however, challenges persist: *T. cruzi* infection in humans remains ethically and technically complex due to chronicity and risk of cardiomyopathy. The suitability of a CHIM is linked to the ability to use a defined infectious dose, a short incubation period, measurable clinical endpoints and effective rescue treatment. Vector‐borne or chronic diseases like Chagas or filariasis may struggle to meet these criteria. Conversely, diseases like hookworm, where larval challenge is feasible and treatment is effective, present a lower barrier to model development. Producing and storing challenge‐grade organisms (e.g. GMP‐grade larvae or parasites) also remains a key hurdle. Novel CHIMs are in development, and a CHIM for Buruli ulcer is likely to begin recruitment and subsequent controlled human infection in the next 2 years.^44^, ^45^

Global research funders such as CEPI, WHO and the Wellcome Trust are increasingly recognising the strategic value of CHIMs in accelerating vaccine development. This is typified by CEPI offering support for CHIM development for MERS‐CoV,^46^ while WHO's target product profiles now incorporate early human challenge data as part of the possible evidence base before licensure. For NTDs, this allows for the possibility of CHIMs to be integrated into vaccine evaluation frameworks, especially where animal models or field trials are impractical.

## 8 CONCLUSION

Controlled human infection models offer a powerful tool for addressing long‐standing bottlenecks in vaccine development for NTDs. Through the deliberate, safe and ethically overseen infection of participants, CHIMs can generate meaningful insights into early immune responses, pathogen dynamics and intervention efficacy. They enable early down‐and up‐selection of vaccine candidates, define correlates of protection and act as a translational bridge between preclinical work and field trials, particularly valuable where traditional pipelines are slow, costly or impractical. The development of a CHIM for *Leishmania major*, as described in this review, represents a significant milestone in translational NTD research. By combining public engagement, rigorous safety protocols and novel ‐omics, the *Leishmania* CHIM has demonstrated that even complex, vector‐borne and disfiguring diseases can be ethically and scientifically studied through controlled infection. This model has yielded new insights into lesion kinetics suggested and vector‐host‐pathogen interactions, while also serving as a proof of concept for the use of CHIMs in vaccine development for parasitic diseases.

CHIMs not only offer a scientifically robust and ethically viable pathway for accelerating vaccine development but also hold potential for reducing global health inequalities. With rigorous protocols, supported by ethical and regulatory frameworks and tailored for endemic settings, CHIMs can support the development of urgently needed vaccines for many at‐risk populations. As infectious diseases research considers emerging threats, CHIMs anchored in lessons from leishmaniasis represent a promising strategy to lead vaccine innovation for many NTDs.

## CONFLICT OF INTEREST STATEMENT

The author declares that they have no personal or financial conflicts of interest that could potentially bias the results or interpretation of the findings presented in this manuscript.

## Data Availability

The datasets generated, analysed and that support the conclusions of this work are available from the author upon reasonable request, with agreement from the study sponsor where relevant.
